# Supplementary material for: Refining the resolution of the yeast genotype–phenotype map using single-cell RNA-sequencing
Source: eLife. 2025 Jul 28;13:RP93906. doi: 10.7554/eLife.93906 (PMC12303567; doi:10.7554/eLife.93906)
Supplement: Supplementary file 1. — We used these variables as explanatory variables in a linear regression model where the relatedness of the high-coverage cells was the response variable. The high-coverage cells were defined as having a coverage in the top 25% of the distribution (>Q3). The data were normalized before performing the linear regression and the influence of the predictors is ranked in decreasing order from top to bottom of the table. [file elife-93906-supp1.docx]

| Regression predictor | *t* value | *p*-value |
| --- | --- | --- |
| HMM-corrected genotype uncertainty | -9.89 | <2.00E-16 |
| Reference panel strain genome uncertainty | -8.36 | <2.00E-16 |
| Breadth of coverage | -2.66 | 8.04E-3 |
| Number of breakpoints | -2.00 | 4.60E-2 |
| Number of reads | -0.10 | 9.18E-1 |
| Depth of coverage | 0.08 | 9.41E-1 |

**Supplementary table 1 Predictors of the relatedness between high-coverage cells and their closest batch 1 lineage.** We used these variables as explanatory variables in a linear regression model where the relatedness of the high-coverage cells was the response variable. The high-coverage cells were defined as having a coverage in the top 25% of the distribution (>Q3). The data were normalized before performing the linear regression and the influence of the predictors is ranked in decreasing order from top to bottom of the table.
