# Supplementary material for: Refining the resolution of the yeast genotype–phenotype map using single-cell RNA-sequencing
Source: eLife. 2025 Jul 28;13:RP93906. doi: 10.7554/eLife.93906 (PMC12303567; doi:10.7554/eLife.93906)
Supplement: Supplementary file 2. [file elife-93906-supp2.docx]

| **Chromosome** | **QTL position** | **Effect size** | **KEGG gene annotation** (60) |
| --- | --- | --- | --- |
| chr01 | 178191 | -0.00337928 | NA |
| chr02 | 469649 | -0.004014246 | YBR112C |
| chr03 | 271959 | 0.005822769 | YCR093W |
| chr04 | 129666 | -0.003667343 | YDL224C |
| chr04 | 277642 | 0.00314934 | YDL122W |
| chr04 | 551436 | -0.002390928 | NA |
| chr04 | 721119 | 0.003213192 | NA |
| chr05 | 195488 | 0.0041835 | YER020W |
| chr07 | 142006 | 0.004597008 | YGL197W |
| chr07 | 544909 | 0.003707906 | YGR032W |
| chr07 | 960936 | -0.0031564 | YGR234W |
| chr08 | 510386 | 0.000987114 | YHR188C |
| chr10 | 440709 | 0.004797933 | YJL005W |
| chr10 | 660787 | 0.013632744 | YJR127C |
| chr11 | 189721 | 0.003792844 | YKL109W |
| chr11 | 581166 | 0.004065964 | YLL061W |
| chr12 | 362424 | 0.005538545 | YLR115W |
| chr12 | 498456 | 0.011796208 | NA |
| chr12 | 647721 | 0.01117107 | NA |
| chr12 | 801095 | -0.002798822 | YLR309C |
| chr12 | 951031 | 0.00991076 | NA |
| chr13 | 51141 | 0.011146938 | YML120C |
| chr13 | 343663 | 0.009595804 | NA |
| chr14 | 442660 | 0.00010508 | YNL094W |
| chr14 | 478696 | 0.025678619 | YNL079C |
| chr15 | 88067 | 0.002939759 | YOL134C |
| chr15 | 163702 | -0.005493485 | YOL081W |
| chr15 | 471091 | -0.009577858 | NA |
| chr15 | 944240 | -0.004171259 | YOR316C |
| chr16 | 515135 | -0.002926068 | YPL023C |
| chr16 | 726411 | 0.002966014 | YPR084W |

**Supplementary table 2 QTL identified from the bulk fitness and DNA sequencing assays.**
