# Supplementary material for: Refining the resolution of the yeast genotype–phenotype map using single-cell RNA-sequencing
Source: eLife. 2025 Jul 28;13:RP93906. doi: 10.7554/eLife.93906 (PMC12303567; doi:10.7554/eLife.93906)
Supplement: Supplementary file 3. [file elife-93906-supp3.docx]

| **Chromosome** | **QTL position** | **Effect size** | **KEGG gene annotation** (60) |
| --- | --- | --- | --- |
| chr01 | 37255 | 0.002989743 | YAL056W |
| chr02 | 509588 | -0.00570928 | YBR112C |
| chr03 | 204909 | 0.006294488 | NA |
| chr04 | 849797 | 0.002871261 | NA |
| chr04 | 1359610 | -0.003216782 | NA |
| chr05 | 189186 | 0.007163705 | YER020W |
| chr07 | 124719 | 0.004653679 | YGL197W |
| chr07 | 391249 | 0.004234537 | YGL071W |
| chr07 | 972982 | -0.002685337 | YGR234W |
| chr08 | 465706 | 0.00218292 | YHR188C |
| chr10 | 422419 | 0.007224158 | YJL005W |
| chr10 | 657711 | 0.01419846 | YJR127C |
| chr11 | 188878 | 0.005193796 | YKL109W |
| chr11 | 613622 | 0.004810808 | YLL061W |
| chr12 | 498456 | 0.016837383 | NA |
| chr12 | 591551 | -0.005087786 | YLR223C |
| chr12 | 657026 | 0.027398457 | NA |
| chr12 | 951087 | 0.012451375 | NA |
| chr13 | 50890 | 0.010534031 | YML120C |
| chr13 | 331755 | 0.008646562 | NA |
| chr14 | 314157 | 0.004021681 | YNL192W |
| chr14 | 481076 | 0.027917558 | YNL079C |
| chr15 | 73395 | 0.002153585 | YOL134C |
| chr15 | 194853 | -0.008589995 | YOL081W |
| chr15 | 467983 | -0.013085813 | NA |
| chr15 | 602954 | 0.002917765 | NA |
| chr15 | 992166 | -0.002155523 | YOR370C |
| chr16 | 511943 | -0.005435177 | YPL023C |
| chr16 | 720831 | 0.00578315 | YPR084W |

**Supplementary table 3 QTL identified from single cells HMM-corrected genotypes and closest lineage fitness.**
